# Immunity to Aphthous Fever1Abstracted by Dr. Otto Noack, Reading, Pa., from Revue Vétérinaire.

**Published:** 1896-05

**Authors:** 


IMMUNITY TO APHTHOUS FEVER.[1] Dr. Leya (*Berliner Thierartzliche Wochenschrift*, Oct. 17, 1895) states that 145 cattle, composing the whole herd of a farmer, were attacked by violent aphthous fever. It was endeavored to keep free from infection a small farm three kilometres (two English miles) distant. A year later the disease appeared at the small farm and affected the cattle of the principal farm. To shorten the duration of the epizoötic, all animals which were still healthy were inoculated in the mouth by the saliva of the sick. The disease showed itself in most of them in three days; but of the 130 animals which composed the herd, 55 resisted inoculation, and it was stated that they had been attacked the year before. This fact shows that they had remained immune during this time.

[1] Abstracted by Dr. Otto Noack, Reading, Pa., from Revue Vétérinaire. [2] Ibid.

